# *CCDC88C‐FLT3* gene fusion in CD34‐positive haematopoietic stem and multilineage cells in myeloid/lymphoid neoplasm with eosinophilia

**DOI:** 10.1111/jcmm.17143

**Published:** 2022-01-12

**Authors:** Yuya Kurihara, Hideaki Mizuno, Akira Honda, Arika Shimura, Yosei Fujioka, Hiroaki Maki, Mineo Kurokawa

**Affiliations:** ^1^ Department of Hematology and Oncology Graduate School of Medicine The University of Tokyo Tokyo Japan; ^2^ Department of Cell Therapy and Transplantation Medicine The University of Tokyo Hospital Tokyo Japan

**Keywords:** allogeneic haematopoietic stem cell transplantation, CCDC88C‐FLT3, myeloid/lymphoid neoplasms with eosinophilia, stem and multilineage cells, tyrosine kinase inhibitor

## CONFLICT OF INTEREST

The authors declare no competing financial interests.

## AUTHOR CONTRIBUTIONS

**Yuya Kurihara:** Formal analysis (lead); Investigation (lead); Methodology (lead); Validation (lead); Writing – original draft (lead). **Hideaki Mizuno:** Methodology (supporting); Project administration (supporting); Supervision (supporting); Writing – review & editing (lead). **Akira Honda:** Conceptualization (supporting); Project administration (supporting); Writing – review & editing (supporting). **Arika Shimura:** Investigation (supporting); Writing – review & editing (lead). **Yosei Fujioka:** Supervision (supporting); Writing – review & editing (supporting). **Hiroaki Maki:** Supervision (supporting); Writing – review & editing (supporting). **Mineo Kurokawa:** Conceptualization (supporting); Investigation (supporting); Methodology (supporting); Project administration (supporting); Supervision (lead); Writing – review & editing (supporting).

To the Editor,

Rearrangements of *PDGFRA*, *PDGFRB*, *FGFR1* or *JAK2* are established features of myeloid/lymphoid neoplasms with eosinophilia (MLN‐Eo).^1^ The rearrangement of the fms‐related tyrosine kinase 3 (*FLT3)* gene should also be associated with MLN‐Eo, and *ETV6*, *SPTBN1*, *GOLGB1* and *TRIP11* have been identified as *FLT3* rearrangement partner genes^2^ (Figure S1). Cases of MLN‐Eo with *FLT3* rearrangement are rare but have a poor outcome.

We encountered a patient who achieved a favourable long‐term outcome by allogeneic haematopoietic stem cell transplantation (allo‐HSCT) and without using tyrosine kinase inhibitors, despite being refractory to conventional chemotherapy. The coiled‐coil domain containing an 88C (*CCDC88C*)‐*FLT3* translocation was identified in this patient who was diagnosed with myeloid neoplasm with T‐cell lymphoblastic lymphoma (T‐LBL). Chronic myelomonocytic leukaemia (CMML) was one of the differential diagnoses for the current patient; the criteria of chronic myelomonocytic leukaemia included not having the specific genes, such as *PDGFRA*, if eosinophilia was present.^1^ The current case showed a *FLT3* rearrangement, and therefore we considered a diagnosis of MLN‐Eo as reasonable. The *CCDC88C‐FLT3* translocation was identified in T‐LBL, CD34‐positive haematopoietic stem and multilineage cells.

## 1 CASE

A 50‐year‐old woman was admitted to our hospital. Her bone marrow aspiration showed hypercellular marrow (>90% cellularity) with increased myeloid cell numbers and abundant eosinophils (10%–20% all nucleated bone marrow cells (Figure S2A)). In addition, T‐LBL was detected in a tonsil biopsy. Tonsil biopsy showed areas with abnormal proliferating lymphoblasts and immunohistochemical findings revealed that abnormal lymphocytes were positive for CD3, CD5, CD7, CD4, CD8, CD56, TdT, CD99 and bcl‐2. The CD4/CD8 ratio was high. Since eosinophils in tonsil specimen were not so condense as in bone marrow, the presence of MLN‐Eo cells were unclear. *JAK2* (V617F), *FLT3‐ITD* (exon 11, 12), *KIT* (D816V) and major/minor *BCR/ABL1* mutations were negative by PCR. A complete blood count showed a white blood cell count of 63.6 × 10^9^/L (neutrophils 20%, lymphocytes 1%, monocytes 59.5%, eosinophils 12%, blasts 3%), haemoglobin 13.3 g/dL and platelets 190 × 10^9^/L. It was not clear whether T‐LBL cells were present in blood or not. G‐banded karyotyping of the bone marrow and tonsils revealed 46, XX, *t*(13;14)(q12;q32) (Figure S2B). Positron emission tomography‐computed tomography examination revealed generalized lymphadenopathy with a maximum standardized uptake value of 5 (Figure S2C).

Assuming that allo‐HSCT would be necessary, conventional chemotherapy was started. The first line regimen was an intensive acute lymphoblastic leukaemia protocol.^3^ The patient's clinical course is shown in Figure S2D. The disease was strongly refractory to conventional chemotherapy. We therefore biopsied the right groin lymph node when it regrew after second‐line chemotherapy (Hyper‐CVAD) and detected not only T‐LBL cells but also MLN‐Eo cells (Figure S2A). G‐banded karyotyping revealed additional chromosomal abnormalities 46, XX, *t*(3;12)(q21;q22), *t*(13;14)(q12;q32) in 2 out of 20 cells.

The first relapse occurred after the consolidation chemotherapy in first‐line therapy (first‐line therapy included the induction and consolidation chemotherapy). Following the second relapse, we chose third‐line therapy using the same regimen as the induction chemotherapy in first‐line therapy. We also planned allo‐HSCT from a human leucocyte antigen–full‐match unrelated donor. The conditioning regimen included etoposide, cyclophosphamide and whole‐body irradiation of 12 Gy. Engraftment took place 22 days after the transplant day. The patient achieved a complete response, with G‐banded karyotyping of bone marrow showing 46, XY without *t*(13;14)(q12;q32) translocation, indicating 100% donor signals. We regularly checked positron emission tomography‐computed tomography, the pathology evaluation and G‐banded karyotyping of bone marrow after transplantation, and confirmed no signs of malignancy for >2 years.

## 2 CCDC88C‐FLT3 REARRANGEMENT

**FIGURE 1:**
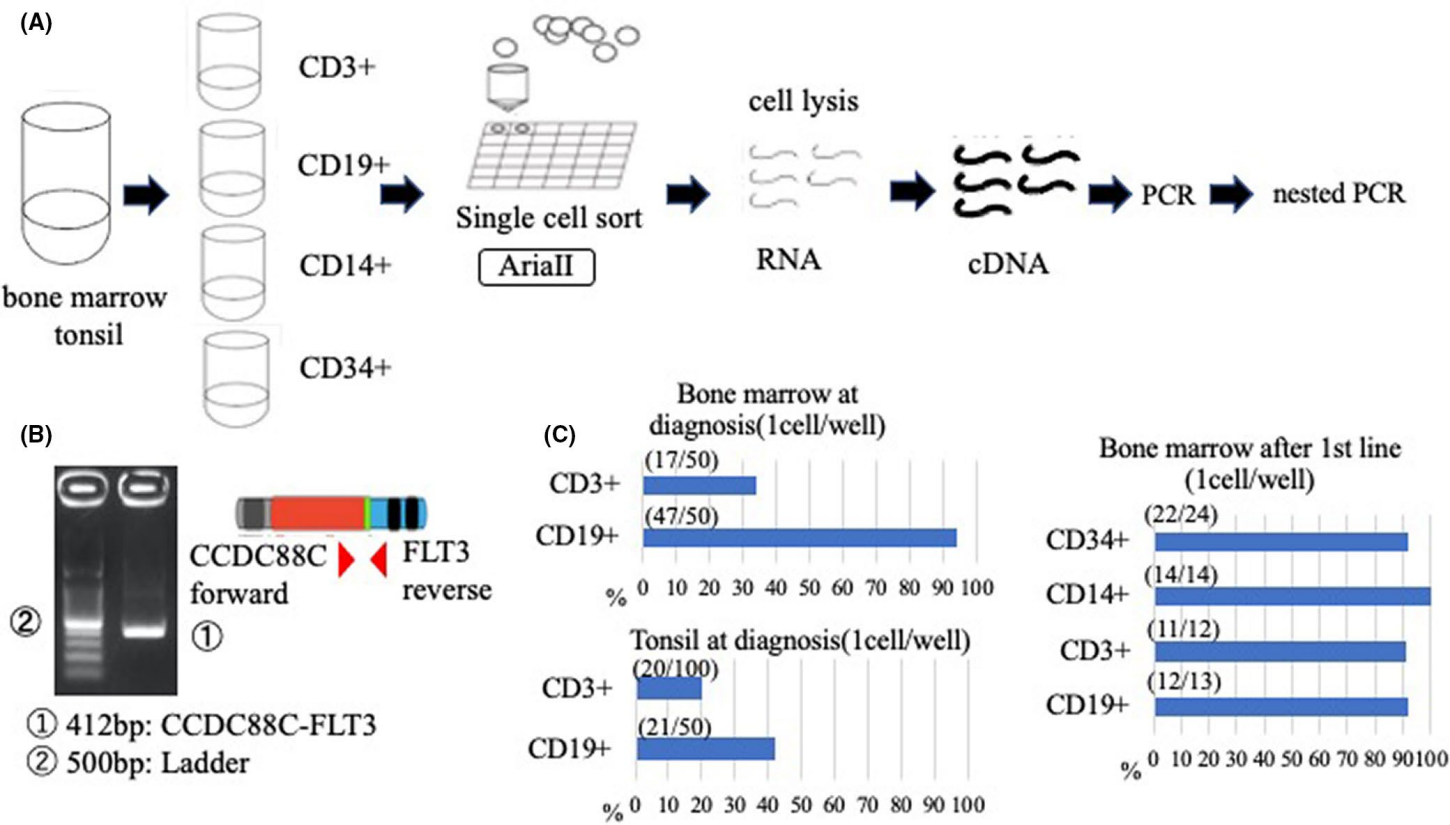
*CCDC88C‐FLT3* breakpoints and occurrence in myeloid and lymphoid lineages. (A) Schema of single‐cell sorting for nested PCR. (B) Electrophoresis of nested PCR products on a 2% agarose gel with bone marrow at diagnosis. RT‐PCR primers were designed to detect 412‐bp products of *CCDC88C‐FLT3* respectively. Lane A: *CCDC88C‐FLT3*; lane C: ladder. Schematic of the position of primers for (C). Red arrows indicate forward and reverse primers for *CCDC88C‐FLT3*. (C) The result of nested RT‐PCR to each single cell. The positive wells are wells in which we could find 969‐bp products of *CCDC88C‐FLT3*. For example, we checked 50 wells after CD3+ single‐cell sorting and found 17 positive wells respectively

We speculated that *FLT3* was associated with the *t*(13;14)(q12;q32) translocation. Previous reports indicated *FLT3* breakpoints in the narrow areas on exon 13, 14 and 15 between the TK domain and juxtamembrane area (Figure S1). Therefore, we investigated the breakpoint using inverse RT‐PCR (Table S1). We first identified the breakpoint using cDNA (Figure 1A,B) and then determined the precise breakpoint using DNA (Figure 1B). Sequencing of the junction using cDNA revealed that one nucleotide was deleted (Figure S1B) through splicing from RNA to cDNA. We specified the *CCDC88C* gene on chromosome 14 and the precise breakpoint. Band q12 on chromosome 13 was thus identified as corresponding to *FLT3* and band q32 on chromosome 14 was identified as *CCDC88C*. While one study reported that *CCDC88C* was a fusion partner gene to *PDGFRB*,^4^ *CCDC88C‐FLT3* has only previously been reported in one case of juvenile myelomonocytic leukaemia in a 20‐week‐old boy.^5^ Therefore, this is the first case of MLN‐Eo with *CCDC88C*‐*FLT3* translocation.

The *CCDC88C* breakpoint in the current case and previous report^5^ were located in the intron after exon 22 and intron after exon 23 respectively. The *FLT3* breakpoint was located in exon 14. The *FLT3* breakpoints in the current and previously reported translocations are all located near exon 14 (Figure S1).

We next examined if the identified translocation occurred only in MLN‐Eo cells or if it also occurred in other lineage cells. Sufficient transcript levels of the *CCDC88C‐FLT3* fusion gene were present in sorted single cells to detect by electrophoresis when amplified by RT‐PCR (Figure 1A‐C and Table S1). The nested RT‐PCR results for each single cell are shown in Figure 1C. The *CCDC88C‐FLT3* breakpoint was amplified in all lineages, indicating the presence of the translocation in both myeloid and lymphoid lineages. The *FLT3* rearrangement thus occurred in CD34‐positive haematopoietic stem cells that differentiated into multiple lineages. The possibility that nested PCR‐negative T‐LBL cells did not include the translocation was low, given that the previous study reported that a mouse model of MLN‐Eo with *FGFR1* rearrangement showed myeloid/lymphoid neoplasms.^6^ The function of *CCDC88C* is not completely known. Daple (encoded by *CCDC88C*) modulates Wnt signalling and leads to the activation of noncanonical Wnt signalling.^7^, ^8^ Tyrosine kinases and Akt are also associated with the signalling. Bio informatic analysis^9^ showed that the *CCDC88C* expression level enriched in Wnt signalling was positively correlated with CD4+ T cell activation. Our patient had the *FLT3* rearrangement which involved tyrosine kinase domain and the T‐LBL which was positive for CD4 strongly. Tyrosine kinase domain is the key factor for MLN‐Eo. Wnt signalling may relate to the emergence of T‐LBL.

In the current report, we identified *CCDC88C* as a novel fusion partner gene to *FLT3*, with the translocation occurring in CD34‐positive haematopoietic stem cells that subsequently differentiate into multiple lineages. The patient achieved a favourable prognosis with allo‐HSCT without TK inhibitor. Although CMML with eosinophilia was one of the differential diagnoses for the current patient, who had a high count of monocytes in the peripheral blood, the FLT3 rearrangement that indicates MLN‐Eo does not lead to the diagnosis of CMML.^1^, ^2^, ^10^, ^11^ MLN‐Eo with *FLT3* rearrangement should thus be included in the World Health Organization definition of MLN‐Eo. Despite the rarity of MLN‐Eo with FLT3 rearrangement, the poor outcome of these patients in the absence of allo‐HSCT highlights the need to investigate the disease aetiology and to develop suitable treatments.

This study was performed in accordance with the Declaration of Helsinki, and informed consent was obtained from the patient for publication of this report.

## Supporting information

App S1

## Data Availability

The data that supports the findings of this study are available in the supplementary material of this article.
