# Supplementary material for: CCDC88C‐FLT3 gene fusion in CD34‐positive haematopoietic stem and multilineage cells in myeloid/lymphoid neoplasm with eosinophilia
Source: J Cell Mol Med. 2022 Jan 12;26(3):950–2. doi: 10.1111/jcmm.17143 (PMC8817136; doi:10.1111/jcmm.17143)
Supplement: Supplementary file 1 — App S1 [file JCMM-26-950-s001.doc]

**Figure S1**

**
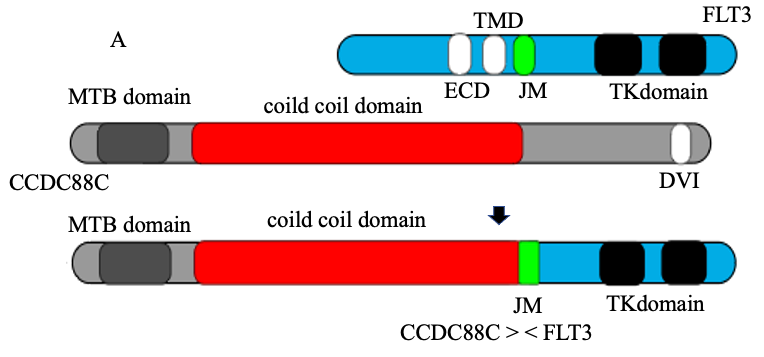
**
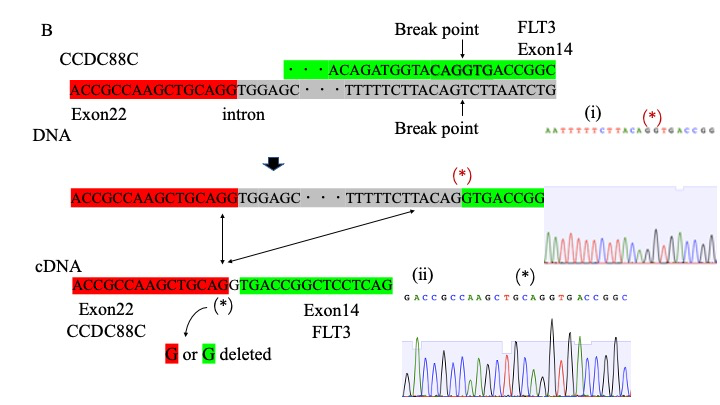


C

**
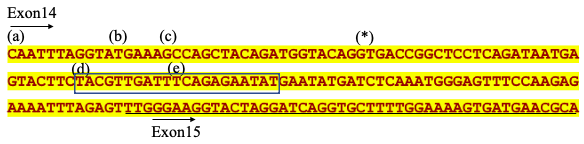
**

**Figure S1**. **The location of break points on *CCDC88C*-*FLT3*.**

(A) Schematic of *FLT3* (top), *CCDC88C* (middle), and the *CCDC88C-FLT3* fusion (bottom). (B) Breakpoint of translocation in *CCDC88C* and *FLT3* (top) in DNA (middle) and cDNA synthesized from RNA (bottom). Red and grey shading indicate exon 22 and intron of *CCDC88C*, respectively; green shading indicates exon 14 of *FLT3.* Sanger sequencing of the breakpoint in DNA (i) and cDNA (ii). One nucleotide, G, was missing from cDNA sequence compared with the genomic DNA. (C) The location of breakpoints on *FLT3*. There are three previous reports on (a) (Ref. 1, 2, 3) which is exon 12. The other points, (b) (c) (d) (e), have one case (Ref. 1, 3, 4, 5) respectively. The current case is on (*). Yellow and bold nucleotide sequences mean the exons. The blue rectangular area is the juxtamembrane autoregulatory region. Underlined sequences indicate the protein kinase domain. Sequence was downloaded from Ensembl at <http://www.ensembl.org/> on 10/10/2020. The location of *FLT3* and *CCDC88C* were NC_000013.11 and NC_000014.9. DVI, dishevelled-binding domain; ECD, extracellular domain; JM, juxtamembrane domain; MTB, putative microtubule-binding domain; TMS, transmembrane domain; TK, tyrosine kinase.

**Ref.**

1. Chonabayashi K, Hishizawa M, Matsui M, Kondo T, Ohno T, Ishikawa T, et al. Successful allogeneic stem cell transplantation with long-term remission of ETV6/ FLT3-positive myeloid/lymphoid neoplasm with eosinophilia. *Ann Hematol*. 2014;**93**(3):535-537.

2. Grand FH, Iqbal S, Zhang L, Russell NH, Chase A, Cross NC. A constitutively active SPTBN1-FLT3 fusion in atypical chronic myeloid leukemia is sensitive to tyrosine kinase inhibitors and immunotherapy. *Exp Hematol*. 2007;**35**(11):1723-1727.

3. Vu HA, Xinh PT, Masuda M, Motoji T, Toyoda A, Sakaki Y, et al. FLT3 is fused to ETV6 in a myeloproliferative disorder with hypereosinophilia and a t(12;13)(p13;q12) translocation. *Leukemia.* 2006;**20**(8):1414-1421.

4. Chung A, Hou Y, Ohgami RS, Gehr AV, Fisk DG, Roskin KM, et al. A novel TRIP11-FLT3 fusion in a patient with a myeloid/lymphoid neoplasm with eosinophilia. *Cancer Genet*. 2017;**216-217**:10-15.

5. Troadec E, Dobbelstein S, Bertrand P, Faumont N, Trimoreau F, Touati M, et al. A novel t(3;13)(q13;q12) translocation fusing FLT3 with GOLGB1: toward myeloid/lymphoid neoplasms with eosinophilia and rearrangement of FLT3? *Leukemia.* 2017;**31**(2):514-517.

**Figure S2**

**Figure S2. Clinical features of the patient.** (A) Tonsil and bone marrow biopsies at diagnosis, and lymph node biopsy at the time of recurrence. Slides of bone marrow biopsy (upper row) show hypercellular marrow (>90% cellularity) with increased myeloid cell numbers and abundant eosinophils (10%–20% all nucleated bone marrow cells, red arrows). CD3- and TdT-stained slides of tonsil biopsy (upper row, ×40 and ×400) showing marked proliferation of tumor cells. Lymph node biopsy (middle row) shows mainly MPO-positive cells, but not T or B cells, indicating that the right groin lymph node was invaded by tumor cells from the myeloid neoplasm with eosinophilia. Flow cytometry showed aberrant expression of T-LBL cells. The CD4/CD8 ratio was high and TdT-stained cells indicated the existence of T-cell lymphoblastic lymphoma cells, they were not dominant at recurrence. (B) G-banded karyotype showing t(13;14) translocation. (C) Positron emission tomography–computed tomography results at diagnosis. Abnormal uptake was confirmed at cervical, upper clavicle, mediastinal, abdominal aortic, ilial and inguinal lymph nodes. (D) Clinical timeline with results of G-banded karyotyping. AraC, cytarabine; BM, bone marrow; BMT, bone marrow transplantation; CPM, Cyclophosphamide; DEX, dexamethasone; DNR, daunorubicin; ETP, etoposide; L-ASP, L-asparaginase; LN, lymph node; MPO, myeloperoxidase; PSL, prednisolone; TdT, terminal deoxynucleotidyl transferase.

**Table S1**

| **Restriction Enzyme** | | **NaeI, EcoRV** | |
| --- | --- | --- | --- |
| **Inverse PCR primer name** | **Sequence** | **Genome DNA primer name** | **Sequence** |
| Nested Rv | GTCCTTCTCCAGCTCCTTC | CCDC88C-forward | GCAGCCAGATCTTGACACTGC |
| Rv | CTTGCTTGGTGAGGTCCCGG | FLT3-reverse | GGAAGGTACTAGGATCAGGTGC |
| For | GCATGCAAGGACACTGACAA |  |  |
| Nested for | GAGGGAGGACCTGGTGCTC |  |  |
| **PCR primer name after single cell sorting** | | **Sequence** | |
| CCDC88C-forward single cell | | GGAGCTTCTCCGAGTGAAG | |
| CCDC88C-forward single cell nested | | CCATCGAGCTGGAGCGGAAT | |
| FLT3-reverse single cell | | ATTCTCGTGGCTTCCCAGCT | |
| FLT3-reverse single cell nested | | GCCTCTCTTTCAGAGCTGTCTGC | |

**Table S1. Restriction enzyme and primer sequences for Inverse PCR and single cell sorting.**

Methods: RNA and DNA obtained from bone marrow aspirates with NucleoSpin® and NucleoSpin® DNA RapidLyse (Takara), then double strand cDNA was synthesized with PrimeScript™ Double Strand cDNA Synthesis Kit (Takara) from RNA. Inverse PCR was performed with restriction enzymes (NaeI and EcoRV) and DNA Ligation Kit Ver.1 (Takara) for self-circularization. The fusion breakpoint identified by genome sequencing was refined by PCR and nested PCR amplification and sequencing. Following agarose gel electrophoresis, the specificity of any observed band was confirmed by sanger sequencing. After the identification of the breakpoint with double strand cDNA, we validated it with DNA by nested PCR and sanger sequencing.

Samples were preserved in the deep freezer and used after thawing.
